# Do Multi-Omics Approaches Improve the Diagnosis of Microbial Overgrowth Syndromes?

**DOI:** 10.1007/s11894-026-01057-y

**Published:** 2026-09-07

**Authors:** Juliana de Freitas Germano, Gabriela Leite, Mark Pimentel

**Affiliations:** 1 Medically Associated Science and Technology (MAST) Program, Cedars- Sinai, Los Angeles, CA USA; 2 Karsh Division of Gastroenterology and Hepatology, Department of Medicine, Cedars-Sinai, Los Angeles, CA USA

**Keywords:** Small intestinal bacterial overgrowth, Intestinal methanogen overgrowth, Intestinal sulfide overproduction, Breath test, Microbiome, Omics

## Abstract

**Purpose of Review:**

This review investigates how advances in breath testing (BT), small bowel (SB) culture, metagenomics, metatranscriptomics, transcriptomics and proteomics are reshaping the definition and diagnosis of small intestinal bacterial overgrowth (SIBO). It also discusses whether SIBO should be redefined as part of a larger group of microbial overgrowth syndromes.

**Recent Findings:**

Recent studies identify distinct hydrogen-, methane-, and hydrogen sulfide-associated overgrowth phenotypes, termed SIBO, intestinal methanogen overgrowth (IMO), and intestinal sulfide overproduction (ISO). SB sampling shows that these conditions involve different microbial patterns and functional activity, symptoms, and host responses. Quantitative shotgun metagenomics provides greater taxonomic and functional resolution than culture, while metatranscriptomics reveals active microbial pathways. On top of that, host transcriptomics and proteomics contribute to the better understanding of the predominant microbial effects in host cellular mechanisms in each of the distinct small bowel overgrowth types.

**Summary:**

SIBO has been increasingly identified as a disorder of microbial ecology and function rather than bacterial quantity alone. Integrating BT with SB sampling and multi-omics approaches may improve classification, clarify symptom mechanisms, and support a more individualized treatment, although standardized methods and further clinical validation remain necessary.

## Introduction

Small intestinal bacterial overgrowth (SIBO) is a condition that has been recognized for over 50 years [1, 2]. Earlier, the classical definitions of SIBO involved a patient with diarrhea, bloating and other symptoms consistent with malabsorption. However, the scenario was often attributed to iatrogenic situations (blind loop syndrome, antrectomy, Bilroth II, among others) where the underlying change in anatomy could account for part of the malabsorption [3, 4]. Yet, it was common to find elevated coliforms in the small bowel (SB) and patients with a combination of SIBO-associated symptoms and positive bacterial culture to respond, in part, to antibiotic therapy [5]. Since that time, SIBO has changed dramatically. With the advent of novel breath testing (BT) [6–10], improved culture techniques [11–13], metagenomic sequencing [12], and other advanced molecular biology techniques [14], we have improved our understanding of the SB ecology. This understanding has now reached the point where the term "SIBO" is not comprehensive enough. In this review, we will tackle the nuances of modern SIBO and describe the evolving science and nomenclature in the context of advanced techniques. 

### What’s in a name? Modern Issues with Diagnostics for Identifying Abnormalities in the SB Microbiome

Given the latest data, there is a need to evolve the concept of SIBO, which was classically believed to be the overgrowth of coliforms (and by inference, colonic flora) in the SB. This inference led to the false notion that colonic bacteria were migrating into the SB and blooming. This is now known to be far from the truth. In fact, with improved culture techniques and next generation sequencing, it has been shown that SIBO is an accumulation of predominantly *Escherichia coli* and *Klebsiella pneumoniae* [12], which belong to the phylum Pseudomonadota (formerly Proteobacteria), one of the two dominant phyla in the SB [15, 16]. This demonstrates that the overgrowth in SIBO supports the role of intrinsic SB bacteria. Later in this paper, it will be discussed how modern techniques are correcting the false previous notions about SIBO.

Defining SIBO remains a challenge. First, it is important to avoid pitfalls with definitions for other related conditions. For example, irritable bowel syndrome (IBS) was originally defined as a clinical diagnosis based on the presence of change in bowel function and the presence of abdominal pain or discomfort [17, 18]. This definition has created tremendous problems in modern medicine because these clinical criteria miss the mark of a gold standard. For example, most patients with active Crohn’s disease meet the Rome criteria. Additionally, most scientists would argue that IBS is likely to be a group of yet undefined and heterogeneous conditions. Some causes of IBS have already been supported by data such as bile acid diarrhea [19], mast cell activation [20], and even SIBO itself [21, 22]. Therefore, defining a disease based on vague clinical criteria is a stumbling block for diagnostic tests if the eventual causes are numerous. Fortunately, SIBO does not suffer from this fate, but it does struggle for a gold standard for benchmark testing.

Several hurdles make identifying a gold standard for SIBO challenging. BT has been a mainstay for detecting SIBO. Originally, BT only included measuring hydrogen (H_2_) on breath after ingestion of a carbohydrate [6, 7, 23]. A rise from baseline of H_2_ on breath within a specified time was considered indicative of SIBO. As designations for SIBO varied by country and paper [24], the first consensus was published in 2017 to standardize the definitions going forward [25]. The consensus on BT has now become global [26, 27]. However, in the late 1990’s, exhaled methane (CH_4_) measurement was also added to BT [8], and hydrogen sulfide (H_2_S), another microbially-produced gas, was included and has been part of the test in the last 5 years [9, 10, 28, 29]. To this point, we have been referring to SIBO as “SIBO”. However, based on contemporary understanding derived from both BT and metagenomic sequencing, there is evidence now that the term “SIBO” may not fully represent all microbial overgrowth types and need nomenclature revision.

Currently, the modern breath test identifies three types of overgrowth in the bowel. These different types are based on overgrowth not only in the SB but also in the colon for some subtypes. The new three-gas breath test is evolving the term “SIBO”, and it is now subdivided into three terms. The first term or type of overgrowth is the more traditional SIBO, whereby the patient has diarrhea and bloating, and H_2_ rises by more than 20 ppm from baseline by 90 min after ingesting the carbohydrate (lactulose or glucose). The second is intestinal methanogen overgrowth (IMO) as defined by the SIBO ACG Guidelines [30]. In this case the patient’s breath demonstrates a CH_4_ level ≥ 10ppm at any point during the test. Methane is associated with bloating, but constipation is the main associated bowel habit [31, 32]. H_2_S, the newest addition to BT, is associated with more severe diarrhea, pain, and bloating, and a recent study showed that intestinal sulfide overproduction (ISO) is supported when the H_2_S is ≥ 2 ppm any time during the breath test [10, 29].

There has been skepticism with BT for being inaccurate and showing low sensitivity and specificity [33], but there are a number of reasons why those criticisms are unfounded. First, defining a gold standard for SIBO is challenging. SB culture was traditionally considered the gold standard for SIBO [34], but recent data question this assumption [16, 35, 36]. As discussed later in the text, quantitative culture requires careful standards, double lumen sterile catheters, and a dedicated lab willing to quantitate SIBO based on a threshold of ≥ 10^3^ CFU/mL (the most recent standard for SIBO) [25–27]. As an example, it has been shown that using a single lumen catheter can result in contamination during SB aspiration for culture at rates as high as 20% of patients’ samples [35], demonstrating that this approach does not meet the criteria for an ideal gold standard. Additionally, an abnormal culture in the absence of symptoms is also unhelpful. The situation is further clouded by the three distinct gasotypes of overgrowth (SIBO, ISO and IMO) on BT: these three gases - H_2_, H_2_S, and CH_4_, respectively - have distinct symptom phenotypes and different microbial profiles with their own particular culture requirements [10]. Thus, the diagnosis of SIBO should really be a combination of compatible symptoms - dominated by bloating - in association with an objective test such as breath test, quantitative culture, or quantitative sequencing (Table 1), which determines the type of overgrowth. Stronger evidence for diagnosing microbial overgrowth would include improvement in symptoms and test results after antibiotic treatment; therefore, sensitivity and specificity do not easily apply here.

**Table 1:** Why determining the sensitivity and specificity of tests for overgrowth is irrational

Criteria for Overgrowth	Rationale
Symptom-based diagnosis	-Symptoms vary by the type of overgrowth [10] -Although bloating is typically ubiquitous to overgrowth, it occurs with many intestinal disorders [10] -Symptoms alone (even with empiric antibiotic use) are not sufficient to diagnose overgrowth
Breath Testing	-Gasotype correlates with symptoms [10] -Positive test along with appropriate symptoms is a good diagnostic evidence of overgrowth [10, 25]
Culture of the small bowel aspirate	-The technique is inconsistent between different centers [12, 27] -It is prone to contamination with single-lumen catheter [35] -Positive culture (≥ 10^3^ CFU/mL) combined with appropriate symptoms is a good evidence of overgrowth [25, 26]
Quantitative metagenomic sequencing of the small bowel aspirate	-It shows better reproducibility than culture [37] -Quantity of microbes correlates with symptoms [12] -Identifies the three current microtypes of overgrowth [37]
Metatranscriptomics of the small bowel aspirate	-Indirectly measures the overgrowth based on prevalence of microbial metabolic pathways [38]
Transcriptomics of the small bowel mucosa	-Measures the host consequences of overgrowth [14] -The area is currently nascent but has high potential
Proteomics of the small bowel aspirate	-Investigates the proteomics profile of secreted proteins [39] -Identifies cellular host responses to overgrowth -It is a nascent area

Original table

### Breath Tests in Identifying Microbial Overgrowth

BT is a simple non-invasive technique for assessing microbial overgrowth. As mentioned above, current BT can investigate up to three gases (H_2_, CH_4_, and H_2_S) that have different implications and identify SIBO, IMO and ISO [10, 28], respectively. A large prospective cohort study of an at home breath test showed that SIBO (H_2_) is associated with diarrhea, while CH_4_ is associated with constipation, and H_2_S, the new gas, is related to greater diarrhea, pain, urgency and overall severity [10].

The association of CH_4_ with constipation has been known for some time [37]. Since this initial description, it has been shown that CH_4_ in fact causes a slowing of the GI tract [38, 39], and that treating methanogens with a combination of antibiotics such as rifaximin and neomycin reduces methane and, consequently, improves constipation [40]. In addition, the level of CH_4_ on breath test also correlates with the level of the methanogen *Methanobrevibacter smithii* in the stool, and subjects with constipation-predominant IBS (IBS-C) frequently have CH_4_ on the breath [28]. Further, another recent study demonstrated that breath CH_4_ correlates with *M. smithii* levels in aspirates of the SB as well [29].

In the case of H_2_S, recent data have shown that levels of this gas are higher in IBS with diarrhea [28] and, overall, predict higher severity of diarrhea and pain [10]. These findings suggest BT to assess all three gases is important to understand the underlying phenotype and the underlying microbiome, as well.

### From Stool Microbiome Studies to the SB: Why Location Matters

Over the past two decades, advances in next-generation sequencing have transformed microbiome research. Since the publication of the Human Microbiome Project, thousands of studies have investigated associations between the gut microbiome and a wide range of human diseases, including obesity, diabetes, IBD, colorectal cancer, neurological disorders, and IBS [41, 42]. Despite this remarkable growth in the field, reproducible associations between individual microbial species and specific diseases remain relatively uncommon. Instead, most disorders appear to be characterized by shifts in microbial communities, ecological networks, or functional capacity rather than the presence or absence of a single pathogen.

One important explanation for this limited reproducibility is that the overwhelming majority of microbiome studies have relied on stool samples. While fecal samples are easily obtained and adequately represent the distal colonic microbiota, they provide only an indirect view of microbial communities inhabiting other regions of the gastrointestinal tract. Recent work has demonstrated that the gastrointestinal tract is composed of multiple distinct microbial ecosystems that differ substantially in bacterial biomass, oxygen tension, nutrient availability, bile acid concentrations, antimicrobial peptide exposure, and host immune interactions, resulting in marked regional differences in microbial composition and function [42, 43].

The SB represents one of the most distinctive microbial environments within the gastrointestinal tract. Unlike the densely populated anaerobic ecosystem of the colon, the SB contains a lower microbial biomass enriched in facultative anaerobic organisms adapted to rapidly fluctuating environmental conditions. Studies using optimized aspiration techniques combined with high-throughput sequencing have shown that the normal SB microbiota differs substantially from stool and is characterized by enrichment of Pseudomonadota and Bacillota (formerly Firmicutes), whereas many obligate anaerobes that dominate fecal communities are comparatively underrepresented [12, 15, 16, 44].

This distinction has major implications for SIBO research. Because bacterial overgrowth occurs within the SB, stool microbiome analyses may fail to identify the microorganisms and microbial interactions directly responsible for symptom generation. Accordingly, direct investigation of the SB microbiome has become increasingly recognized as essential for understanding the pathophysiology of SIBO and developing more accurate diagnostic approaches.

### SB Culture: the Historical Gold Standard

For decades, quantitative culture of SB aspirates has been regarded as the reference method for diagnosing SIBO because it directly measures viable bacterial growth within the SB [1, 25, 45]. Historically, bacterial concentrations exceeding 10⁵ colony-forming units (CFU)/mL diagnosed SIBO; however, more recent evidence has demonstrated that bacterial counts ≥ 10³ CFU/mL are associated with significant alterations in microbial ecology and gastrointestinal symptoms, leading current North American and international consensus guidelines to adopt this lower threshold [25, 26].

Although culture has served as the historical gold standard, several limitations restrict its diagnostic performance. Only a subset of intestinal microorganisms can be cultivated under conventional laboratory conditions, particularly fastidious and oxygen-sensitive bacteria. Also, bacterial recovery is highly dependent on aspiration techniques, transport conditions, culture media, incubation parameters, and laboratory expertise, contributing to substantial variability among studies [27]. Contamination during sample collection also remains a significant concern, with rates approaching 20% when single-lumen aspiration catheters are used [35], as mentioned above.

Perhaps the greatest limitation of culture is that it only measures cultivable bacterial abundance while providing relatively little information regarding microbial diversity, ecological interactions, or metabolic function. Consequently, modern molecular approaches have increasingly supplemented or potentially may replace culture by providing a more comprehensive characterization of the SB microbiome.

### The Evolution of SB Microbiome Profiling: from 16S rRNA Sequencing to Quantitative Shotgun Metagenomics

The introduction of culture-independent sequencing technologies fundamentally transformed microbiome research. Initially, 16S ribosomal RNA (rRNA) gene sequencing became the predominant approach for characterizing bacterial communities because it enabled identification of microorganisms independent of culture [41]. This technology greatly expanded our understanding of microbial diversity and revealed that the human gastrointestinal tract harbors a substantially more complex microbial ecosystem than previously appreciated.

Despite its major contributions, 16S sequencing possesses several important limitations. Taxonomic resolution is often limited to the genus level, amplification bias may influence relative abundance estimates, and the technique provides only proportional representation of bacterial communities rather than absolute bacterial load [46]. These limitations are particularly relevant for SIBO, where disease is fundamentally defined by bacterial overgrowth rather than alterations in relative microbial composition.

Whole-genome shotgun metagenomic sequencing addressed many of these shortcomings by sequencing all the microbial DNA present within a sample. Compared with 16S sequencing, shotgun metagenomics enables species- and frequently strain-level taxonomic identification while simultaneously provides information regarding bacterial, archaeal, viral, and fungal communities as well as their functional genetic potential [47].

More recently, quantitative shotgun metagenomics has further advanced microbiome characterization by integrating sequencing with measurements of total microbial biomass, thereby allowing estimation of the absolute abundance of individual microbial species rather than their relative abundance alone [48]. This quantitative approach is particularly attractive for diseases such as SIBO, where microbial load itself represents the defining pathological feature.

### Quantitative Shotgun Metagenomics: Toward a New Reference Standard for SIBO?

Among emerging molecular approaches, quantitative shotgun metagenomics appears particularly promising for the evaluation of SIBO. Unlike conventional culture, which is restricted to cultivable organisms, quantitative shotgun sequencing simultaneously quantifies total microbial burden, identifies microorganisms at species-level resolution, and characterizes the functional genetic potential of the entire microbial community.

Recent work by Leite and colleagues demonstrated that quantitative sequencing of duodenal aspirates closely paralleled quantitative culture while providing substantially greater biological resolution. Importantly, sequencing identified marked reductions in microbial diversity, disruption of microbial ecological networks, and expansion of specific *Escherichia coli* and *Klebsiella pneumoniae* strains that strongly correlated with abdominal pain, bloating, and diarrhea severity [12]. In addition, microbial pathways involved in carbohydrate fermentation, hydrogen production, and hydrogen sulfide metabolism were enriched in patients meeting the contemporary SIBO threshold of ≥ 10³ CFU/mL, suggesting that sequencing provides mechanistic insights that extend beyond bacterial enumeration alone [12].

These observations raise an important question: should quantitative shotgun metagenomics become the new reference standard for SIBO? An ideal diagnostic method should accurately quantify bacterial burden, comprehensively characterize microbial composition, demonstrate high reproducibility across laboratories, correlate with symptom severity, and predict treatment response. Quantitative shotgun sequencing fulfills many of these criteria while overcoming several inherent limitations of culture. Nevertheless, widespread clinical implementation will require standardized sampling protocols, harmonized bioinformatic pipelines, validated diagnostic thresholds, and reductions in sequencing cost before routine adoption becomes feasible.

At present, quantitative shotgun metagenomics should be viewed as complementary rather than competitive with culture. Culture remains valuable because it confirms microbial viability and permits downstream phenotypic testing, whereas quantitative sequencing provides a comprehensive assessment of microbial abundance, taxonomic composition, and functional potential. As molecular technologies continue to evolve, quantitative shotgun metagenomics will likely become the preferred research standard for characterizing small intestinal microbial overgrowth and may ultimately serve as the molecular reference standard for SIBO diagnosis.

Although quantitative shotgun metagenomics represents a major advance over both culture and conventional sequencing approaches, it remains fundamentally a measure of microbial abundance and genetic potential rather than microbial function. Even when absolute bacterial load is determined, shotgun sequencing identifies which microorganisms and genes are present, but it does not establish whether those genes are actively being expressed or contribute to disease pathophysiology [47, 49]. This distinction is particularly important in SIBO, where symptom generation, including gas production, carbohydrate fermentation, bile acid metabolism, and interactions with the intestinal epithelium are driven by microbial metabolic activity rather than microbial presence alone. Consequently, two patients with similar microbial composition and bacterial burden may exhibit markedly different clinical presentations because their microbiota differs in functional activity. Recent studies of the SB microbiome further support this concept by demonstrating that quantitative microbial burden and microbial composition correlate with symptoms but still do not fully explain the biological mechanisms underlying distinct overgrowth phenotypes [12, 28]. In this context, metatranscriptomic studies become key.

### Metatranscriptomics: How Can This Be Helpful in SIBO Diagnosis?

Metatranscriptomics, since its first description in 2005 [50], has evolved as a high throughput shotgun sequencing method that provides information to perform functional studies and examine the total microbiome’s active biological pathways. Opposite to metagenomics, which determines microbial taxonomy or composition, metatranscriptomics investigates what genes are actively expressed in the microbiome, as it is related to the sequencing of microbial mRNA instead of DNA. Results obtained from metatranscriptomics studies have shown more associations with symptoms and different disease states than those from shotgun metagenomics. In a study that was part of the Integrative Human Metagenomics Project in IBD, for example, findings revealed that during disease flare-ups, the metagenome remained relatively stable while the metatranscriptomics results shifted. Results showed that *Alistipes putredinis*, which monopolized the methylerythritol phosphate pathway transcriptionally, recruited *Bacteroides vulgatus* in this same pathway during flare-ups, while the metagenomics results remained constant longitudinally in one participant of the study; also, some species that were metagenomically abundant were in fact dormant, such as *Dialister invisus*, while others were more active but less abundant, such as *B. vulgatus*, which had predicted pathways more abundant in non-IBD controls metagenomically, but showed those same pathways substantially more active in IBD at the RNA level [51]. In IBS, stool metatranscriptomics seems to shift according to the subtypes, and IBS-D shows activation of transcripts associated with fructose and mannose metabolism, polyol utilization, and other fermentable-carbohydrate-processing functions vs. healthy controls; additionally, IBS-C differs from IBS-D primarily at the activity level instead of the taxonomic signature, with less transcriptional upregulation of carbohydrate-utilization and fermentation pathways [52].

Most subjects with IBS-D also present SIBO [22, 53] which, as mentioned, can be detected by increased levels of exhaled breath H_2_ [25]. One study showed that the H_2_-producing and SIBO-associated family Enterobacteriaceae [12] was correlated with the ISO-associated *Fusobacterium* and *Desulfovibrio* genera - which are known H_2_S producers - in stool samples from IBS-D subjects; conversely, specific H_2_-producing bacterial families, such as Ruminococcaceae and Christensenellaceae, were linked to an increased RA of the Methanobacteriaceae family [28], which includes the methane producer *M. smithii*, closely associated with IBS-C [28] and IMO [29]. This way, H_2_-producing microbes may associate with distinct microbial networks, and this may depend on the still unknown gene activity pattern of these microbial populations in the SB. Also, fecal samples may not be representative of the SIBO-related microbial functional level.

To overcome the lack of information regarding metatranscriptomics in bacterial overgrowth, our group has shown in preliminary data that more than 91% of the differentially expressed bacterial genes in SB aspirates from SIBO vs. non-SIBO participants were upregulated and associated with “metabolic pathways” and “biosynthesis of secondary metabolites” [54], which closely relate to the formation of gases. Therefore, it is important to note the impact that the SB metatranscriptomics would have on qualifying - and not only quantifying, as in metagenomics - bacterial overgrowth, as the usage of microbial genes may help point to mechanisms of disease, show direct association with symptomatology, identify other possible microbial players, and be determinant of treatment approach, especially when SIBO co-occurs with ISO and/or IMO.

As our understanding of SIBO evolves from a condition defined by bacterial overgrowth to one characterized by microbial ecology and metabolism, metatranscriptomics has the potential to provide the critical missing component needed to connect microbial composition with function and, ultimately, clinical phenotype.

### SB Gene and Protein Expression: What Do We Know About Host Transcriptomics and Proteomics in SIBO?

On top of microbial composition and functional activity, host transcriptomics and proteomics analyses become crucial to determining gut host-microbiome interactions in health and disease, as bacterial products can directly affect the host. In IBS, most human studies up to date involve the use of colonic tissue or colonic luminal content to determine changes in transcriptomics profiles, but symptomatology could be potentially explained by alterations in the SB function [55]. A few studies that used PCR panels to investigate gene expression of the SB mucosa in IBS demonstrated upregulation of genes associated with barrier function, such as *TJP1* and claudins [56, 57], while genes associated with immune function were downregulated, such as *IL-1B* and *CCL20* [56]. When considering IBS-D vs. IBS-C, transcriptomics data analyzed by Camilleri and colleagues did not show relevant results in SB tissue [58], whereas our group identified several changes in gene expression that were related with alterations in redox balance, IBS-D pathophysiology, ATP synthesis, mitochondrial respiration, water homeostasis, ferroptosis, pain, and gut motility in SB biopsies from subjects with ISO; importantly, these findings in ISO differed from those in IMO and SIBO [14]. Combined, these data may reflect the need to consider IBS and its underlying mechanisms in sample grouping, as distinct bacterial overgrowth may dictate changes in transcriptomics and, possibly, other omics results. Moreover, the lack of similarities in transcriptomics profiles from the different gasotype groups [14] suggest that subjects with SIBO, ISO and IMO may benefit from different treatment approaches.

Studies that involve the SB have relied on techniques other than proteomics, such as western blot and immunohistochemistry, to investigate a small panel of host mucosal proteins associated with mechanisms in IBS, including those related to tight junctions, immune response, and ion channels [56, 57, 59]. While this approach is relevant and can contribute to the development of strategies for treatment, a broader understanding of all potential pathways involved in gut-associated disorders could be more informative. Recent preliminary data from our group with a larger cohort of SIBO vs. non-SIBO (independent of IMO/ISO diagnosis) showed several differentially expressed secreted proteins that suggest upregulation of oxidative species, ferroptosis and host defense involving defensins as part of SIBO when proteomics of the SB luminal aspirates are considered [60]. On the other hand, we have also demonstrated that transcriptomics changes in the duodenal tissue from a smaller cohort of SIBO vs. non-SIBO subjects (excluding concurrent IMO and/or ISO) were unexpressive but involved downregulation of genes such as the *POM121* and *ZP3* fusion (*POMZP3*), the transport and Golgi organization protein 6 (*TANGO6*), and the hyaluronan synthase 3 (*HAS3*), which is associated with inflammatory response [14]. It is important to note that the mechanisms identified in the proteomics of the SB aspirates in SIBO reflect some of those identified in the transcriptomics of ISO [14, 60]. Thus, while group size is known to interfere with results, once again the gasotypes may have a central role in shifting transcriptomics and proteomics findings in SB disorders such as IBS and bacterial overgrowth. This is corroborated, at least in part, by the fact that rats demonstrated distinct SB transcriptomics profiles when grouped according to their predominant gut bacterial microtype after cytolethal distending toxin B inoculation in a model of IBS-D like phenotypes that included *Escherichia*- and *Desulfovibrio*-predominant animal clusters [61].

**Fig. 1:**
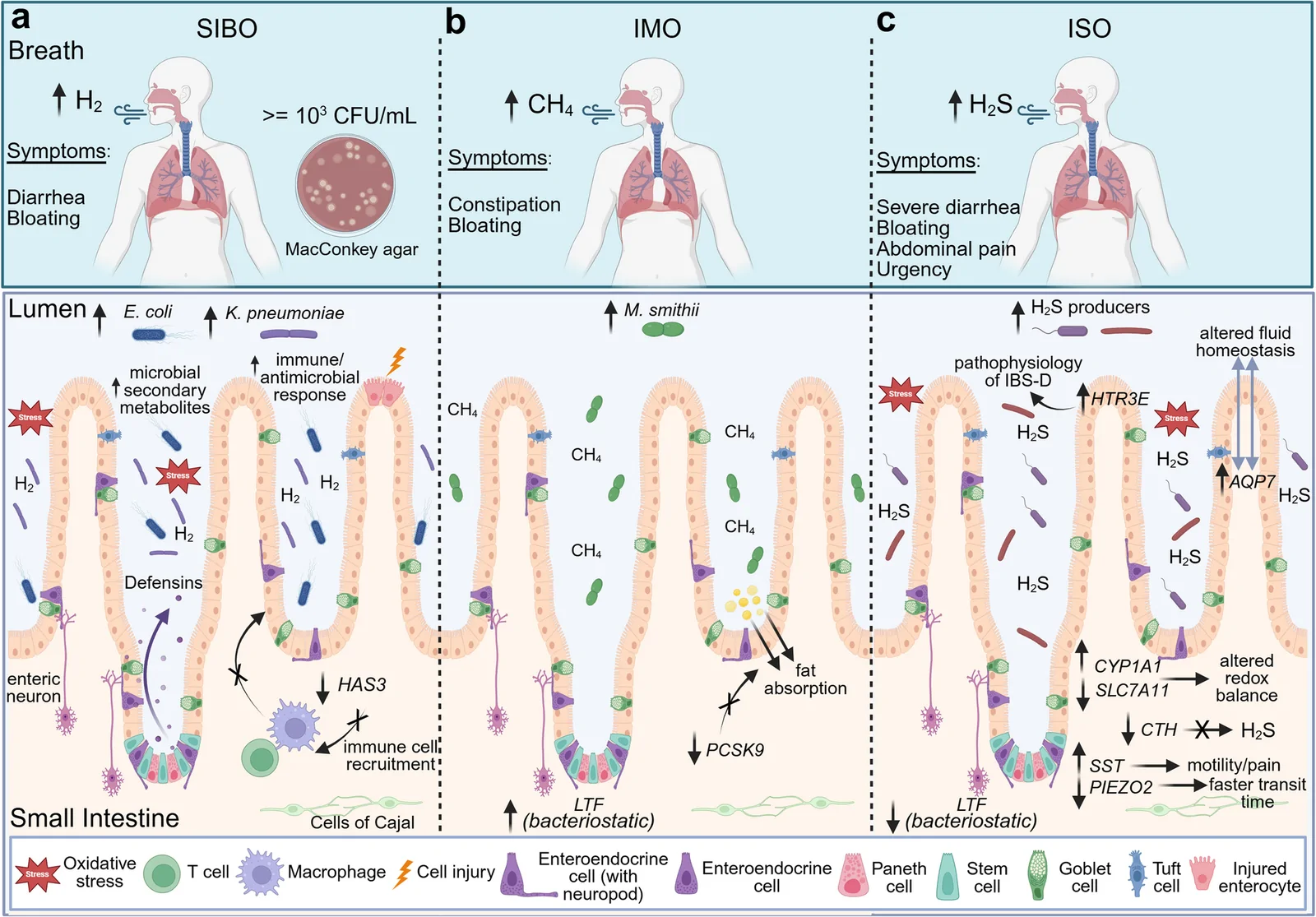
Small intestinal bacterial overgrowth (SIBO), intestinal methanogen overgrowth (IMO), and intestinal sulfide overproduction (ISO) diagnosis and mechanisms. **a**, SIBO has traditionally been defined by elevated small-bowel (SB) bacterial counts, commonly ≥ 10³ CFU/mL in SB aspirates, in patients with compatible symptoms such as bloating and diarrhea. With the broader adoption of breath testing, noninvasive measurement of exhaled hydrogen (H₂) became a practical approach for identifying H_2_-predominant overgrowth. Molecular profiling of duodenal aspirates further links SIBO with expansion of Proteobacteria, particularly *Escherichia coli* (*E. coli*) and *Klebsiella pneumoniae* (*K. pneumoniae*). Preliminary metatranscriptomics of human duodenal aspirates suggest increased microbial metabolic activity, including pathways related to secondary metabolite production, whereas preliminary proteomics of duodenal luminal contents indicates activation of epithelial antimicrobial and oxidative-response programs. Host transcriptomics of duodenal biopsies has also identified altered immune- and barrier-related genes, including downregulation of *HAS3*; **b**, IMO is characterized by elevated exhaled methane (CH₄) and is clinically associated with bloating, constipation, and delayed intestinal transit. This phenotype reflects overgrowth of methanogenic archaea, particularly *Methanobrevibacter smithii* (*M. smithii*), whose abundance correlates with methane production and constipation severity. In duodenal biopsy transcriptomics, IMO has been associated with downregulation of *PCSK9*, a gene implicated in fat absorption, and upregulation of *LTF*, which encodes the bacteriostatic protein lactoferrin. **c**, ISO is characterized by elevated breath hydrogen sulfide (H₂S) and overgrowth of H₂S-producing organisms. Duodenal microbial studies link higher H₂S levels with sulfate-reducing or sulfide-producing taxa. Host transcriptomics from duodenal biopsies shows dysregulation of genes such as the serotonin receptor involved in IBS-D pathophysiology *HTR3E*, and others associated with fluid homeostasis (*AQP7*), motility (*SST*, *PIEZO2*), pain (*SST*), redox balance (*CYP1A1*, *SLC7A11*), endogenous H₂S production (*CTH*), and antimicrobial defense (*LTF*), supporting ISO as a distinct overgrowth phenotype. The figure is original and was created with BioRender.com

Thus, while data regarding transcriptomics and proteomics in SIBO are still exploratory and preliminary, the findings already demonstrate the potential of these next generation techniques in contributing to the mechanisms involved in the development of symptoms. Therefore, there is an unmet need for the use of SB samples, and integration of microbial and host omics data - which is still scarce - for a better understanding of the pathophysiology of gut disorders and host-microbiome interactions. A summary of findings is described in (Fig. 1).

## Conclusions

There is evidence that SIBO is no longer adequately defined as a simple bacterial overgrowth in the SB. Instead, evidence supports the existence of distinct overgrowth categories characterized by gasotypes, microbial communities and functional activity, and host responses. Here, we conclude that direct SB sampling, quantitative metagenomics, metatranscriptomics, transcriptomics, and proteomics provide complementary information that would not be acquired by stool analysis or culture alone. Integrating these techniques may contribute to the improvement of diagnosis, symptomatology, gasotype distinction, and guide treatment approaches. Standardized sampling, analytical methods, and thresholds are, nonetheless, essential for routine clinical practice. Unfortunately, advanced omics strategies remain impractical for clinical practice today. BT remains the most cost-effective test and when combined with symptoms and clinical response may be the best gold standard for microbial overgrowth for at least the near term. 

## Key References

- Pimentel, M., et al., *Real-world Study of Three-gas Breath Testing Nationwide and the Association With Symptoms.* J Clin Gastroenterol, 2026. **60**(5): p. 406–417.
  - ○ Assesses whether three-gas breath testing identifies distinct microbial overgrowth syndromes and their associated gastrointestinal symptoms.
- Leite, G., et al., *Defining Small Intestinal Bacterial Overgrowth by Culture and High Throughput Sequencing.* Clin Gastroenterol Hepatol, 2024. **22**(2): p. 259–270.
  - ○ Example of the use of shotgun metagenomics to improve SIBO diagnostics and to better identify SIBO-associated microbes.
- McCallum, G. and C. Tropini, The gut microbiota and its biogeography. Nat Rev Microbiol, 2024. 22(2): p. 105–118.
  - ○ Discusses the heterogeneity of the microbiome profile in different gut segments.
- de Freitas Germano, J., et al., Transcriptomics profiles in intestinal sulfide overproduction, small intestinal bacterial overgrowth, and intestinal methanogen overgrowth. mSystems, 2026: p. e0045826.
  - ○ Explores differences in the small bowel transcriptomics in SIBO, IMO, and ISO.
- Jacobs, J.P., et al., *Multi-omics profiles of the intestinal microbiome in irritable bowel syndrome and its bowel habit subtypes.* Microbiome, 2023. **11**(1): p. 5.
  - ○ Example of the use of multi-omics microbial signatures that distinguish IBS and its subtypes by integrating microbiome composition, microbial gene expression, and metabolite profiles.

## Data Availability

No datasets were generated or analysed during the current study.
